# The undernourished curriculum: What happened to nutritional education in the medical curriculum?

**DOI:** 10.3389/fnut.2025.1672864

**Published:** 2025-10-15

**Authors:** Katarina Milosavljevic, Jessica Meng, Varsha Sahoo, Kevin Trac, Jelissa Hernandez Lopez, Saranjeet Kaur, Anaha Raghunathan, Kamilah Ali

**Affiliations:** Touro College of Osteopathic Medicine, New York, NY, United States

**Keywords:** nutrition, education, osteopathic, lifestyle medicine, medical students

## Abstract

Nutrition is a cornerstone of disease prevention and health promotion, yet medical education, particularly within osteopathic schools, continues to fall short in preparing future physicians to address nutrition in clinical care. Despite the rising burden of diet-related conditions such as obesity, diabetes, and cardiovascular disease, most United States medical students receive fewer than the recommended 25 h of nutrition education. This perspective article explores the current state of nutrition education in osteopathic medical schools, examines the role of emerging clinical models such as “Food is Medicine,” and outlines opportunities for reform that align with the holistic philosophy of osteopathic medicine.

## Introduction

Nutrition plays a foundational role in promoting health and preventing disease. Leading health organizations, including the American Diabetes Association, American Heart Association AHA, American College of Cardiology, World Health Organization (WHO), and United States (U. S). Preventive Services Task Force, all recognize lifestyle interventions such as dietary counseling, physical activity, and behavioral support as essential first-line strategies for managing chronic conditions. These evidence-based approaches have been shown to improve outcomes in diseases like diabetes, cardiovascular disease, and obesity. The WHO’s Global Strategy on Diet, Physical Activity, and Health emphasizes that chronic diseases account for over 70% of global deaths annually, placing a growing burden on health systems worldwide (1).

In the United States, this burden is particularly acute. Diet-related conditions such as diabetes and obesity have surged in prevalence, disproportionately affecting underserved populations and driving up healthcare costs. In 2022, the annual cost of diabetes reached $412.9 billion, with $106.3 billion lost to reduced productivity (2). Obesity now affects over 40% of adults and 20% of children (3), contributing an estimated $173 billion annually in direct medical expenses (3). Both conditions are major contributors to cardiovascular disease, the nation’s leading cause of death, which costs $229 billion annually and is projected to affect 184 million Americans by 2050 (4). A 2016 global burden of disease analysis attributed more than 5% of risk-attributable disability-adjusted life years to poor diet, underscoring the need to prioritize nutrition within public health and clinical care (5).

Despite the central role of nutrition in chronic disease prevention and management, medical education, particularly within osteopathic programs, continues to inadequately prepare future physicians for this critical area of care. A national survey found that only 45% of U. S. medical schools offer the minimum recommended 25 h of nutrition education (6). A follow-up study of 30 osteopathic schools revealed that only seven provided even a fraction of this training, averaging just 4.1 h (7). Graduate medical education fares no better: 77% of residency directors report their programs lack adequate nutrition training, and only 14% of resident physicians feel prepared to counsel patients (8, 9). Given that medical school may be the only formal exposure many physicians have to nutrition science and counseling, addressing these gaps is both urgent and foundational to improving patient outcomes.

This perspective article examines the current state of nutrition education in osteopathic medical curricula, highlights the critical need for comprehensive nutrition training among current and future medical practitioners, and evaluates proposed improvements to strengthen and standardize curriculum design.

### Current state of nutrition in osteopathic medical schools

Educating the public about nutrition begins with the training of healthcare providers. Despite widespread recognition of nutrition’s role in disease prevention and health promotion, physicians often feel underprepared to provide dietary counseling (10–12). This stems from limited exposure to nutrition in medical education. The National Academy of Sciences recommended in 1985 that medical students receive at least 25 h of nutrition instruction (13), yet a 2008 survey of 109 allopathic schools found an average of only 19.6 h, well below the standard (6). Osteopathic programs reflect a similar trend. A 2015 survey of 30 osteopathic medical schools found that 85% did not meet the 25-h benchmark, and nearly one-third offered less than half that amount (7). A national survey revealed that 78.7% of osteopathic and 90.5% of allopathic students rated their nutrition education as inadequate (14, 15). There are no recent comprehensive surveys published in 2025, and subsequent reviews after 2021 indicate that little progress has been made, with most schools still providing fewer than 25 h of nutrition education (10, 16). This shortfall is especially concerning for osteopathic graduates, many of whom enter primary care fields that emphasize prevention.

Although various institutions have initiated improvements, there remains no unified set of competencies or guidelines to structure nutrition education in osteopathic schools. Interactive and skill-based curricula have been shown to improve retention (17), while interprofessional approaches involving registered dietitians, culinary instructors, and behavioral health specialists enhance clinical relevance (18). However, the impact of these strategies has yet to be systematically evaluated. In a review of 42 U. S. accredited osteopathic colleges’ published websites across 68 campuses, eight out of 21 offered structured nutrition education and six have published outcome data (Table 1). Structured nutrition education are courses formally integrated into the medical curriculum and do not include stand-alone electives or optional workshops. Of these programs the majority rely on brief modules or electives rather than longitudinal integration. This shows that the absence of evaluation methods and national oversight limits scalability. Without consistent infrastructure, faculty development, and curricular alignment, osteopathic students remain variably equipped to provide nutrition-informed care.

**Table 1:** Nutrition programs at osteopathic medical schools.

Osteopathic medical colleges	Program structure	Effectiveness of the program
Alabama College of Osteopathic Medicine	Lifestyle medicine elective.	No publication
Arkansas College of Osteopathic Medicine	GI and Nutritional Health module- four lectures on diets and anatomic issues impacting nutrition.	No publication
AT Still University (Kirksville)	Culinary Medicine elective course featuring dietary journal articles and hands-on experience in a local kitchen.	No publication
AT Still University (SOMA)	Osteopathic Wellness Lifestyle program consists of hands-on culinary workshops.	
Baptist Health Sciences University College of Osteopathic Medicine	Special Topics courses for first- and second-year students.	No publication
California Health Sciences University	Team-based learning, case studies, and hands-on cooking classes. Nutritional education in all courses during preclinical years.	Survey found that baseline diets improved and nutrition knowledge increased after program participation as measured by the Healthy Eating Index (42).
Des Moines University College of Osteopathic Medicine	Healthy Food Preparation Elective with hands-on meal prep.	No publication
Edward Via College of Osteopathic Medicine (Auburn)	Interdisciplinary culinary elective involving online educational modules and hands-on cooking at an on-campus science center for medical, pharmacy, and nutrition students.	Interdisciplinary culinary medicine elective with online education modules, case-based discussions, and hands-on meal preparation significantly improved medical students’ nutrition literacy and confidence in providing nutrition counseling (43).
Idaho College of Osteopathic Medicine	Culinary Medicine elective for second year students with a non-profit community organization and health center.	No publication
Lake Erie College of Osteopathic Medicine	First years take eight modules on Health Meets Food-based elective that blends virtual teaching with live cooking. Third- and fourth-year students complete 8 focused modules in-person or virtually at a community center.	No publication
Meritus School of Osteopathic Medicine	Topics on community health and preventive medicine integrated into preclinical curriculum.	No publication
Nova Southeastern University	Personal wellness course and Health Meets Food modules are available in the first-year curriculum.	No publication
Philadelphia College of Osteopathic Medicine (Pennsylvania)	Four-day elective with virtual nutrition and biochemistry modules. Hands-on cooking classes open to first- and second-year students.	A retrospective cohort study on the culinary curriculum revealed a significant difference in student’s nutritional knowledge and confidence in counseling patients on diets (44).
Rocky Vista University (unsure campus)	Electives focused on implementing nutritional plans for patients.	No publication
Sam Houston State University College of Osteopathic Medicine	Gastrointestinal & Nutrition course in second year. Also teaches the Health meets Food curriculum.	No publication
TouroCOM (Montana)	Three-week program with nutrition didactics and hands-on meal preparation.	No publication
TouroCOM (California)	Nutrition topics in first-year biochemistry and pathology courses. Five mandatory events in preclinical years regarding vitamins, cancer nutrition, cardiovascular disease, and sports medicine.	No publication
TouroCOM (Nevada)	Experimental elective paired eight online didactic sessions on cardiovascular nutrition and counseling materials with nine in-person culinary medicine workshops.	Survey analysis showed students strongly recommend the elective. Third- and fourth-year students reported feeling unprepared in nutrition counseling (45).
University of the Incarnate Word	GI system, Nutrition, and Appetite course.	No publication
West Virginia School of Osteopathic Medicine	Culinary and Lifestyle Medicine program open to medical students of all years with externships, hands-on cooking lessons, and virtual medicine education taught by various medical professionals. Curriculum is part of Health meets Foods. There is also the Culinary and Lifestyle Medicine Track that spans all 4 years and involves online and in-person activities including hands-on teaching kitchens, journal clubs, summer externships, and a capstone project.	A two-week applied learning culinary medical elective improved nutritional knowledge and self-efficacy (46).
William Carey University College of Osteopathic Medicine	First year course on nutrition course designed with registered dieticians, lecturers and an exercise physiologist with tasks including creating recipes and personalized physical fitness plans for patients.	First-year students were more likely to believe that nutritional interventions are key for disease prevention compared to the control group (47).

Table 1 was generated systematically by reviewing the official websites of all 42 accredited U. S. colleges of osteopathic medicine, to identify courses, programs, or other forms of nutrition content incorporated into the medical curriculum. PubMed search was used to identify published outcomes for nutrition education initiatives using key words “osteopathic, nutrition, education, lifestyle medicine and students”. Data obtained directly from medical school published websites as of June 2025.

### Barriers to implementation

Despite attempts to integrate nutrition education in the medical curriculum, there are significant barriers. Funding is a major barrier, especially for experiential programs like culinary medicine that require dedicated space, trained faculty, and supplies. Early efforts, such as the National Institute of Health (NIH) Nutrition Academic Award (1998–2005), provided temporary support but lacked sustained resources (19). Institutions also face logistical challenges in modifying curricula. Adding nutrition content requires faculty buy-in, curricular space, and coordination across departments. Nutrition is often taught in biochemistry or physiology, without explicit clinical context or behavioral applications (10). This limits students’ confidence and perceived relevance. A shortage of qualified faculty compounds the issue. Many schools lack access to registered dietitians or certified nutrition professionals, which limits the quality and scope of instruction (20, 21). Experiential components like problem-based learning or kitchen-based instruction, shown to enhance student engagement, are still underutilized (22). Finally, nutrition remains underemphasized on licensing exams. Without curricular standards or testing benchmarks, institutions may deprioritize it compared to other subjects (23). This creates a feedback loop that reinforces minimal instruction and limited clinical application.

### Food is Medicine: a model for integrating clinical care and education

As healthcare delivery increasingly incorporates food-based clinical interventions, future physicians must be equipped to understand and implement them (24). The emerging “Food is Medicine” (FIM) movement exemplifies how nutrition is no longer a peripheral topic—it is a central component of patient care. Preparing medical students, particularly in osteopathic programs where preventive and holistic care are core values, to engage with FIM principles is essential for ensuring the next generation of physicians can effectively address diet-related chronic disease.

The FIM movement refers to a set of clinical interventions that use food as a formal component of disease treatment and prevention. These include medically tailored meals (MTMs), medically tailored groceries (MTGs), and produce prescription programs (PRx), all of which aim to address chronic disease management, improve nutritional status, and reduce healthcare utilization. These programs exemplify how nutrition can be implemented as structured medical therapy, offering a compelling framework for training future physicians.

- MTMs are fully prepared, condition-specific meals prescribed for patients with complex illnesses. Studies have shown MTMs reduce hospitalizations by 49%, emergency visits by 30%, and overall healthcare costs by 16% (25, 26). In 2023, the Tufts Food is Medicine Institute projected that MTMs could prevent 1.6 million hospitalizations and save $13.6 billion annually if scaled nationally (27).
- MTGs provide raw ingredients aligned with patients’ health needs and cooking capacity. Interventions have demonstrated improvements in glycemic control and systolic blood pressure (28–30), offering a scalable model for patients with lower clinical risk or greater food agency.
- PRx allows healthcare providers to prescribe fruits and vegetables to patients at risk of diet-related illness. Emerging evidence links these programs to improve hemoglobin A1c, body mass index, and blood pressure, as well as enhanced food security and patient satisfaction (29–31). These interventions are often supported by federal and philanthropic funding, including the USDA’s Gus Schumacher Nutrition Incentive Program.

FIM programs also advance health equity by targeting populations disproportionately affected by food insecurity and chronic disease. These models emphasize community-centered care, cultural relevance, and behavioral change, aligning well with osteopathic principles of whole-person medicine.

Despite their clinical promise, the success of Food is Medicine programs ultimately depends on healthcare professionals who are trained to screen for food insecurity, collaborate with nutrition professionals, and integrate dietary interventions into treatment plans. However, most medical students and residents lack formal training in these competencies. As FIM becomes more widely adopted in clinical settings, osteopathic medical education must ensure that students graduate equipped to contribute meaningfully to such models, through competencies in nutrition assessment, patient education, and interprofessional collaboration.

### Opportunities for reform

There are promising opportunities to improve medical nutrition education that can better equip students to guide their patients and empower them to feel confident and competent in their roles as future physicians. Integrating evidence-based nutrition education and practical training into osteopathic and allopathic medical schools’ curricula is key (18).

1. Funding for nutrition education: past, present, and future
  Efforts to fund nutrition education in medical schools have spanned public, private, and institutional levels. One of the earliest initiatives, the Nutrition Academic Award (NAA), was launched in 1998 by the National Heart, Lung, and Blood Institute with support from the National Institute of Diabetes and Digestive and Kidney Diseases. The NAA aimed to integrate nutrition education across 21 U. S. schools through faculty development, online modules, and case-based teaching tools (22). Despite its promise, the program’s five-year funding cap hindered long-term implementation by limiting institutional capacity to retain nutrition experts.
  Between 1991 and 1997, a separate NIH/NCI grant at the University of Arizona resulted in a 115% increase in nutrition instruction hours and significantly improved student satisfaction. Even after the grant concluded, the school retained a 57% increase in baseline instruction, highlighting the need for sustainable external support (32).
  More recently, the American Association of Colleges of Osteopathic Medicine launched the Food as Medicine initiative in 2024. This program awarded grants to osteopathic medical schools with a focus on interprofessional collaboration, experiential learning, and curriculum design in nutrition. These efforts represent a significant step toward institutionalizing nutrition education, though funding across institutions remains fragmented and often time-limited.
  Future funding strategies could involve partnerships with organizations such as the American College of Lifestyle Medicine and the Academy of Nutrition and Dietetics. Medical schools might also allocate continuing medical education budgets toward nutrition training or seek philanthropic support from donors focused on chronic disease prevention. Finally, aligning nutrition education with broader public health efforts could help secure additional resources and elevate its clinical relevance.
2. Integrative approaches for medical nutrition education
  To embed nutrition into clinical training, medical schools must adopt practical, evidence-based models. Team-based learning (TBL) has proven to improve understanding and application of nutritional principles through active engagement, problem-solving, and peer discussion (32–35). TBL reduces reliance on faculty while encouraging accountability and retention.
  Interprofessional education also plays a key role. In clinical practice, nutrition care often involves collaboration with Registered Dietitian Nutritionists, Certified Nutrition Specialists, and other allied professionals. These experts are infrequently included in medical education, despite their relevance to patient-centered care (36). Involving them in curriculum design enhances both content and application.
  Experiential learning, especially kitchen-based instruction, builds clinical confidence. Programs like Cooking for Health Optimization with Patients, which combine hands-on culinary education with Mediterranean diet counseling, led to an 82% increase in students’ likelihood of adopting and recommending the diet compared to peers in traditional training (22). These models offer a pragmatic approach to empowering osteopathic physicians to integrate nutrition into patient care.
3. Nutrition and Medical Licensure
  Requiring nutrition education as part of medical licensure would ensure all physicians receive standardized, evidence-based training. This mirrors previous reforms that integrated ethics and psychiatry into licensing exams such as the USMLE and COMLEX (37, 38). A national consensus has already outlined core nutrition competencies and an interprofessional roadmap spanning UME, GME, CME, demonstrating the feasibility of embedding nutrition within assessment framework (39). Including nutrition in board assessments through dedicated questions or case-based scenarios would signal its clinical relevance, encourage curricular investment, and promote accountability across institutions.
4. Lifestyle Medicine and Broader Health Integration

The emerging field of lifestyle medicine extends the “Food is Medicine” framework by incorporating nutrition, physical activity, stress management, and behavioral counseling to prevent and treat chronic disease (40, 41). The American College of Lifestyle Medicine offers board certification and educational pathways to equip providers with holistic, non-pharmacologic strategies that align with osteopathic principles.

## Conclusion

Improving nutrition education in osteopathic medical schools is no longer simply optional but rather essential for the future. As chronic diseases linked to poor diet continue to rise, future physicians must be equipped with the knowledge and skills to provide nutrition-informed care. While many institutions recognize the importance of this training, agreeing with the value of nutrition and prevention is not enough. Systemic action is needed to translate these values into educational reform. Medical schools must invest in faculty development, infrastructure, and curriculum integration to ensure students are prepared to address nutrition in clinical practice. Establishing national standards, incorporating nutrition into licensing exams, and supporting experiential, interprofessional models of instruction are critical next steps. By aligning educational priorities with evolving clinical demands, osteopathic medical education can lead the way in preparing physicians to deliver whole-person, preventative care grounded in nutrition science.

## Data Availability

The original contributions presented in the study are included in the article/supplementary material, further inquiries can be directed to the corresponding author.
